# What is context in knowledge translation? Results of a systematic scoping review

**DOI:** 10.1186/s12961-024-01143-5

**Published:** 2024-04-29

**Authors:** Tugce Schmitt, Katarzyna Czabanowska, Peter Schröder-Bäck

**Affiliations:** Department of International Health, Care and Public Health Research Institute – CAPHRI, Faculty of Health, Medicine and Life Sciences, Maastricht University, Maastricht, The Netherlands

**Keywords:** Knowledge Translation, Innovation, Evidence-informed policymaking, Context, Health systems

## Abstract

Knowledge Translation (KT) aims to convey novel ideas to relevant stakeholders, motivating their response or action to improve people’s health. Initially, the KT literature focused on evidence-based medicine, applying findings from laboratory and clinical research to disease diagnosis and treatment. Since the early 2000s, the scope of KT has expanded to include decision-making with health policy implications.

This systematic scoping review aims to assess the evolving knowledge-to-policy concepts, that is, macro-level KT theories, models and frameworks (KT TMFs). While significant attention has been devoted to transferring knowledge to healthcare settings (i.e. implementing health policies, programmes or measures at the meso-level), the definition of 'context' in the realm of health policymaking at the macro-level remains underexplored in the KT literature. This study aims to close the gap.

A total of 32 macro-level KT TMFs were identified, with only a limited subset of them offering detailed insights into contextual factors that matter in health policymaking. Notably, the majority of these studies prompt policy changes in low- and middle-income countries and received support from international organisations, the European Union, development agencies or philanthropic entities.

## Background

Few concepts are used by health researchers as vaguely and yet as widely as Knowledge Translation (KT), a catch-all term that accommodates a broad spectrum of ambitions. Arguably, to truly understand the role of context in KT, we first need to clarify what KT means. The World Health Organization (WHO) defines KT as ‘the synthesis, exchange and application of knowledge by relevant stakeholders to accelerate the benefits of global and local innovation in strengthening health systems and improving people’s health’ [1]. Here, particular attention should be paid to ‘innovation’, given that without unpacking this term, the meaning of KT would still remain ambiguous. Rogers’ seminal work ‘Diffusion of Innovations’ [2] defines innovation as an idea, practice or object that is perceived as novel by individuals or groups adopting it. In this context, he argues that the *objective* novelty of an idea in terms of the amount of time passed after its discovery holds little significance [2]. Rather, it is the *subjective* perception of newness by the individual that shapes their response [2]. In other words, if an idea seems novel to individuals, and thereby relevant stakeholders according to the aforementioned WHO definition, it qualifies as an innovation. From this perspective, it can be stated that a fundamental activity of KT is to communicate ideas that could be perceived as original to the targeted stakeholders, with the aim of motivating their response to improve health outcomes. This leaves us with the question of who exactly these stakeholders might be and what kind of actions would be required from them.

The scope of stakeholders in KT has evolved over time, along with their prompted responses. Initially, during the early phases of KT, the focus primarily revolved around healthcare providers and their clinical decisions, emphasising evidence-based medicine. Nearly 50 years ago, the first scientific article on KT was published, introducing Tier 1 KT, which concentrated on applying laboratory discoveries to disease diagnosis or treatment, also known as bench-to-bedside KT [3]. The primary motivation behind this initial conceptualisation of KT was to engage healthcare providers as the end-users of specific forms of knowledge, primarily related to randomised controlled trials of pharmaceuticals and evidence-based medicine [4]. In the early 2000s, the second phase of KT (Tier 2) emerged under the term ‘campus-to-clinic KT’ [3]. This facet, also known as translational research, was concerned with using evidence from health services research in healthcare provision, both in practice and policy [4]. Consequently, by including decision-makers as relevant end-users, KT scholars expanded the realm of research-to-action from the clinical environment to policy-relevant decision-making [5]. Following this trajectory, additional KT schemes (Tier 3–Tier 5) have been introduced into academic discourse, encompassing the dissemination, implementation and broader integration of knowledge into public policies [6, 7]. Notably, the latest scheme (Tier 5) is becoming increasingly popular and represents the broadest approach, which describes the translation of knowledge to global communities and aims to involve fundamental, universal change in attitudes, policies and social systems [7].

In other words, a noticeable shift in KT has occurred with time towards macro-level interventions, named initially as evidence-*based* policymaking and later corrected to evidence-*informed* policymaking. In parallel with these significant developments, various alternative terms to KT have emerged, including ‘implementation science’, ‘knowledge transfer’, and ‘dissemination and research use’, often with considerable overlap [8]. Arguably, among the plethora of alternative terms proposed, implementation science stands out prominently. While initially centred on evidence-based medicine at the meso-level (e.g. implementing medical guidelines), it has since broadened its focus to ‘encompass all aspects of research relevant to the scientific study of methods to promote the uptake of research findings into routine settings in clinical, community and policy contexts’ [9], closely mirroring the definition to KT. Thus, KT, along with activities under different names that share the same objective, has evolved into an umbrella term over the years, encompassing a wide range of strategies aimed at enhancing the impact of research not only on clinical practice but also on public policies [10]. Following the adoption of such a comprehensive definition of KT, some researchers have asserted that using evidence in public policies is not merely commendable but essential [11].

In alignment with the evolution of KT from (bio-)medical sciences to public policies, an increasing number of scholars have offered explanations on how health policies should be developed [12], indicating a growing focus on exploring the mechanisms of health policymaking in the KT literature. However, unlike in the earlier phases of KT, which aimed to transfer knowledge from the laboratory to healthcare provision, decisions made for public policies may be less technical and more complex than those in clinical settings [3, 13, 14]. Indeed, social scientists point out that scholarly works on evidence use in health policies exhibit theoretical shortcomings as they lack engagement with political science and public administration theories and concepts [15–18]; only a few of these works employ policy theories and political concepts to guide data collection and make sense of their findings [19]. Similarly, contemporary literature that conceptualises KT as an umbrella term for both clinical and public policy decision-making, with calls for a generic ‘research-to-action’ [20], may fail to recognise the different types of actions required to change clinical practices and influence health policies. In many respects, such calls can even lead to a misconception that evidence-informed policymaking is simply a scaled-up version of evidence-based medicine [21].

In this study, we systematically review knowledge translation theories, models and frameworks (also known as KT TMFs) that were developed for health policies. Essentially, KT TMFs can be depicted as bridges that connect findings across diverse studies, as they establish a common language and standardise the measurement and assessment of desired policy changes [22]. This makes them essential for generalising implementation efforts and research findings [23]. While distinctions between a theory, a model or a framework are not always crystal-clear [24], the following definitions shed light on how they are interpreted in the context of KT. To start with, theory can be described as a set of analytical principles or statements crafted to structure our observations, enhance our understanding and explain the world [24]. Within implementation science, theories are encapsulated as either generalised models or frameworks. In other words, they are integrated into broader concepts, allowing researchers to form assumptions that help clarify phenomena and create hypotheses for testing [25].

Whereas theories in the KT literature are explanatory as well as descriptive, KT models are only descriptive with a more narrowly defined scope of explanation [24]; hence they have a more specific focus than theories [25]. KT models are created to facilitate the formulation of specific assumptions regarding a set of parameters or variables, which can subsequently be tested against outcomes using predetermined methods [25]. By offering simplified representations of complex situations, KT models can describe programme elements expected to produce desired results, or theoretical constructs believed to influence or moderate observed outcomes. In this way, they encompass theories related to change or explanation [22].

Lastly, frameworks in the KT language define a set of variables and the relations among them in a broad sense [25]. Frameworks, without the aim of providing explanations, solely describe empirical phenomena, representing a structure, overview, outline, system or plan consisting of various descriptive categories and the relations between them that are presumed to account for a phenomenon [24]. They portray loosely-structured constellations of theoretical constructs, without necessarily specifying their relationships; they can also offer practical methods for achieving implementation objectives [22]. Some scholars suggest sub-classifications and categorise a framework as ‘actionable’ if it has the potential to facilitate macro-level policy changes [11].

Context, which encompasses the entire environment in which policy decisions are made, is not peripheral but central to policymaking, playing a crucial role in its conceptualisation [26–34]. In the KT literature, the term ‘context’ is frequently employed, albeit often with a lack of precision [35]. It tends to serve as a broad term including various elements within a situation that are relevant to KT in some way but have not been explicitly identified [36]. However, there is a growing interest in delving deeper into what context refers to, as evidenced by increasing research attention [31, 32, 37–41]. While the definition of context in the transfer of knowledge to healthcare settings (i.e. implementing health policies, programmes or measures at the meso-level) has been systematically studied [36, 37, 42, 43], the question of how KT scholars detail context in health policymaking remains unanswered. With our systematic scoping review, we aim to close this gap.

## Methods

While KT TMFs, emerged from evidence-based medicine, have historically depicted the use of evidence from laboratories or healthcare organisations as the gold standard, we aimed to assess in this study whether and to what extent the evolving face of KT, addressing health policies, succeeded in foregrounding ‘context’. Our objective was thus not to evaluate the quality of these KT TMFs but rather to explore how scholars have incorporated contextual influences into their reasoning. We conducted a systematic scoping review to explore KT TMFs that are relevant to agenda-setting, policy formulation or policy adoption, in line with the aim of this study. Therefore, publications related to policy implementation in healthcare organisations or at the provincial level, as well as those addressing policy evaluation, did not meet our inclusion criteria. Consequently, given our focus on macro-level interventions, we excluded all articles that concentrate on translating clinical research into practice (meso-level interventions) and health knowledge to patients or citizens (micro-level interventions).

Prior systematic scoping reviews in the area of KT TMFs serve as a valuable foundation upon which to build further studies [44, 45]. Using established methodologies may ensure a validated approach, allowing for a more nuanced understanding of KT TMFs in the context of existing scholarly work. Our review methodology employed a similar approach to that followed by Strifler et al. in 2018, who conducted a systematic scoping review of KT TMFs in the field of cancer prevention and management, as well as other chronic diseases [44]. Their search strategy was preferred over others for two primary reasons. First, Strifler et al. investigated KT TMFs altogether, systematically and comprehensively. Second, unlike many other review studies on KT, they focused on macro-level KT and included all relevant keywords useful for the purpose of our study in their Ovid/MEDLINE search query [44]. For our scoping review, we adapted their search query with the assistance of a specialist librarian. This process involved eliminating terms associated with cancer and chronic diseases, removing time limitation on the published papers, and including an additional language other than English due to authors’ proficiency in German. We included articles published in peer-reviewed journals until November 2022, excluding opinion papers, conference abstracts and study protocols, without any restriction on publication date or place. Our search query is presented in Table 1.

**Table 1:** Ovid/MEDLINE search query used for our systematic scoping review

1	(knowledge adj2 (application or broke$ or creation or diffus$ or disseminat$ or exchang$ or implement$ or management or mobili$ or translat$ or transfer$ or uptak$ or utili$)).tw
2	(evidence$ adj2 (exchang$ or translat$ or transfer$ or diffus$ or disseminat$ or exchang$ or implement$ or management or mobil$ or uptak$ or utili$)).tw
3	(KT adj2 (application or broke$ or diffus$ or disseminat$ or decision$ or exchang$ or implement$ or intervent$ or mobili$ or plan$ or policy or policies or strateg$ or translat$ or transfer$ or uptak$ or utili$)).tw
4	(research$ adj2 (diffus$ or disseminat$ or exchang$ or transfer$ or translation$ or application or implement$ or mobil$ or transfer$ or uptak$ or utili$)).tw
5	("research findings into action" or "research to action" or "research into action" or "evidence to action" or "evidence to practice" or "evidence into practice").tw
6	Diffusion of Innovation/ or (diffusion adj2 innovation*).tw
7	(("systematic review$" or "knowledge synthes$") adj5 ("decision mak$" or "policy mak$" or "policy decision?" or "health polic$")).tw
8	(("systematic review$" or "knowledge synthes$") adj2 (application or implement$ or utili?ation or utilize? or utilise? or utili?ing)).tw
9	research utili?ation.tw
10	((evidence base$ or evidence inform$) adj2 (decision$ or plan$ or policy or policies or practice or action$)).tw
11	or/1–10
12	(health policy or health planning or health plan implementation or health care reform or health services administration).sh. or ((health or healthcare or health care) adj2 (polic$ or plan$ or implement$ or reform$ or administrat$)).ab,ti
13	(theor$ or framework$ or model$ or concept$).ab,ti
14	11 and 12 and 13
15	limit 14 to (english or german)

**Fig. 1:**
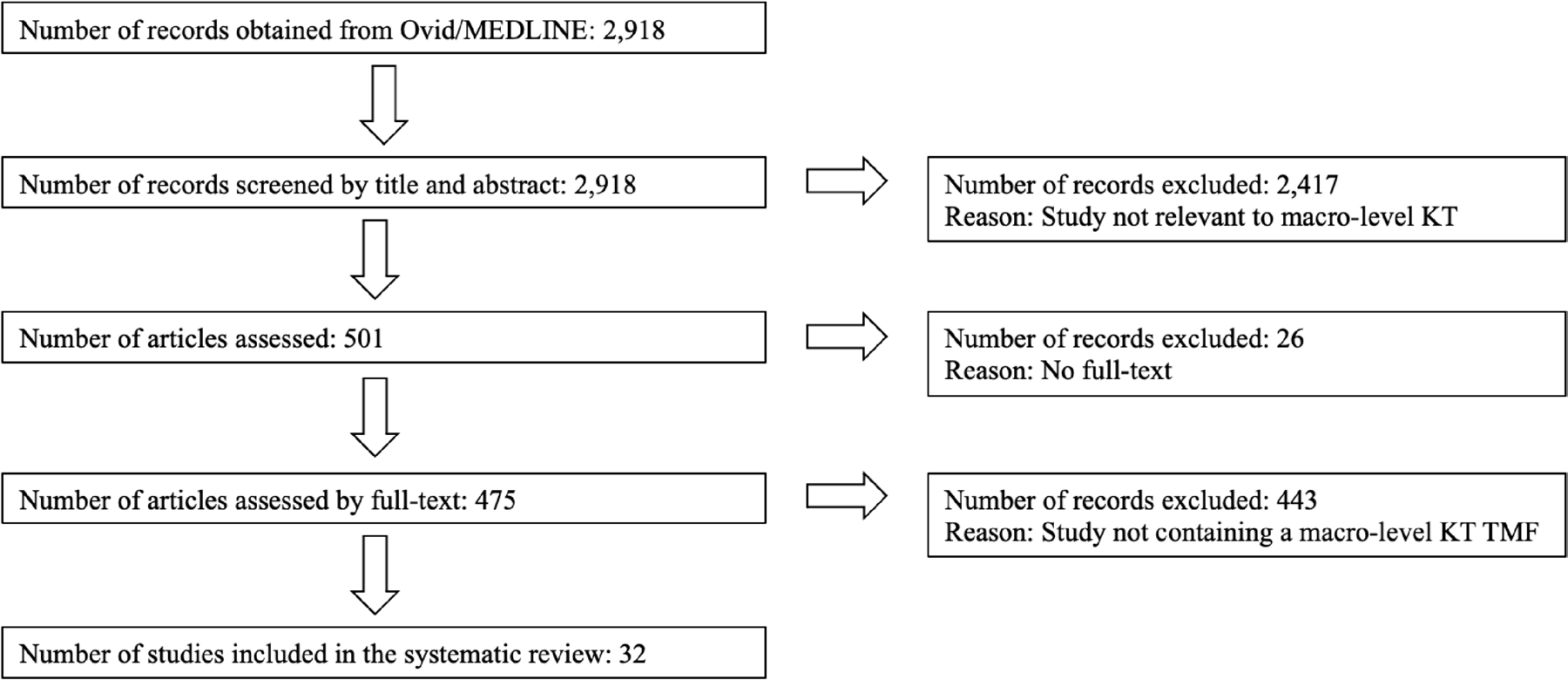
Flow chart of the review process

Following a screening methodology similar to that employed by Votruba et al. [11], the first author conducted an initial screening of the titles and abstracts of 2918 unique citations. Full texts were selected and scrutinised if they appeared relevant to the topics of agenda-setting, policy formulation or policy adoption. Among these papers, the first author also identified those that conceptualised a KT TMF. Simultaneously, the last author independently screened 2918 titles and abstracts, randomly selecting 20% of them to identify studies related to macro-level KT. Regarding papers that conceptualised a KT TMF, all those initially selected by the first author underwent a thorough examination by the last author as well. In the papers reviewed by these two authors of this study, KT TMFs were typically presented as either Tables or Figures. In cases where these visual representations did not contain sufficient information about ‘context’, the main body of the study was carefully scrutinised by both reviewers to ensure no relevant information was missed. Any unclear cases were discussed and resolved to achieve 100% inter-rater agreement between the first and second reviewers. This strategy resulted in the inclusion of 32 relevant studies. The flow chart outlining our review process is provided in Fig. 1.

## Results

According to the results of our systematic scoping review (Table 2), the first KT TMF developed for health policies dates back to 2003, confirming the emergence of a trend that expanded the meaning of the term Knowledge Translation to include policymakers as end-users of evidence during approximately the same period. In their study, Jacobson et al. [46] present a framework derived from a literature review to enhance understanding of user groups by organising existing knowledge, identifying gaps and emphasising the importance of learning about new contexts. However, despite acknowledging the significance of the user group context, the paper lacks a thorough explanation of the authors’ understanding of this term. The second study in our scoping review provides some details. Recognising a shift from evidence-based medicine to evidence-based health policymaking in the KT literature, the article by Dobrow et al. from 2004 [30] emphasises the importance of considering contextual factors. They present a conceptual framework for evidence-based decision-making, highlighting the influence of context in KT. Illustrated through examples from colorectal cancer screening policy development, their conceptual framework emphasises the significance of context in the introduction, interpretation and application of evidence. Third, Lehoux et al. [47] examine the field of Health Technology Assessment (HTA) and its role in informing decision and policymaking in Canada. By developing a conceptual framework for HTA dissemination and use, they touch on the institutional environment and briefly describe contextual factors.

**Table 2:** Results of our systematic scoping review

No	Author, year	Study	KT TMF name	Focus; geographic area	Financial interests	Authors’ definition of ‘context’in their own words
1.	Jacobson et al. 2003	Development of a framework for knowledge translation: understanding user context	Not specified	Not specified; Canada	None declared	No information available
2.	Dobrow et al. 2004	Evidence-based health policy: context and utilisation	Conceptual framework for context-based evidence-based decision-making	Not specified; Canada	HEAL*Net* funded by the Canadian Institutes of Health Research and the Social Sciences and Humanities Research Council of Canada	External contextual factors: • Disease-specific (geographic, demographic and epidemiologic characteristics of a disease), • Extra-jurisdictional • Political (ideological, social, economic and legal) factors
3.	Lehoux et al. 2005	Dissemination of Health Technology Assessments: Identifying the Visions Guiding an Evolving Policy Innovation in Canada	Conceptual Framework of HTA Dissemination and Use	Health Technology Assessment; Canada	Canadian Institutes of Health Research	Institutional environment: • Relationships with other institutional players such as regulatory bodies, knowledge producers, and lobbyists • Established organisational routines • Socio-political context, rules, and resources
4.	Ashford et al. 2006	Creating windows of opportunity for policy change: incorporating evidence into decentralized planning in Kenya	Theorical framework for the transformation of knowledge to policy actions	Health services; Kenya	Bill & Melinda Gates Foundation	No information available
5.	Bauman et al. 2006	Dissemination of Physical Activity Evidence, Programs, Policies, and Surveillance in the International Public Health Arena	Six-Step Framework For International Physical Activity Dissemination	Physical activity; Canada, USA, Brazil, global	None declared	Barriers and facilitators to the PAPH innovation, as part of the six-step model for disseminating international PAPH work: • Barriers: competing public health priorities other than PAPH (such as infections disease control) or competition for resources within broad NCD prevention (such as resources for tobacco or injury control); also consider local cultural and economic factors • Facilitators: a supportive policy framework that provides resources for dissemination of new PAPH approaches
6.	Gold, 2009	Pathways to the Use of Health Services Research in Policy	Factors, Processes, and Actors That Shape Pathways between Research and Its Use	Not specified; USA	Agency for Healthcare Research and Quality	Other influences in process
7.	Green et al. 2009	Diffusion Theory and Knowledge Dissemination, Utilization, and Integration in Public Health	Utilization-focused surveillance framework	Not specified; not specified	None declared	Social determinants/context: • Structures • Economics • Politics • Culture
8.	Dhonukshe-Rutten et al. 2010	European micronutrient recommendations aligned: a general framework developed by EURRECA	General Framework of and for EURRECA	Nutrient requirement; European countries	Commission of the European Communities, Specific Research Technology and Development (RTD) Programme Quality of Life and Management of Living Resources within the 6th framework programme	Socio-political context: • Political and social priorities • Legal context • Ethical issues • Economic implications
9.	Ir et al. 2010	Translating knowledge into policy and action to promote health equity: The Health Equity Fund policy process in Cambodia 2000–2008	The 4-K framework	Equitable health care financing; Cambodia	WHO and POVILL project (Protecting the rural poor against the economic consequences of major illness) funded by the Sixth Framework Programme of the European Commission	No information available
10.	Atun et al. 2010	Integration of targeted health interventions into health systems: a conceptual framework for analysis	Conceptual framework for analysing integration of targeted health interventions into health systems	Complex innovations; not specified	Imperial College London and The World Bank	Context: • Broad context: interplay of the demographic, economic, political, legal, ecological, socio-cultural (including historical legacies), and technological factors in the environment in which the foregoing considerations (the problem, intervention, health system characteristics and the adoption system) • Critical events (such as regime change or a catastrophe) and technological change (such as a new diagnostic tool, a new and affordable drug, or a new prevention mechanism) • Factors beyond the health system (e.g., fiduciary requirements imposed on donor agencies by their governing structures which require them to ‘ring fence’ funding streams or be able to attribute results to their investments or the complexity of fiscal relationships among levels of government, as between central, provincial and local governments in some federal systems)
11.	Bissell et al. 2011	Analysing policy transfer: perspectives for operational research	Model for analysis of health policy transfer	Tuberculosis; Mexico	None declared	Context: • Health system • Political, • Economic, • Social, • Cultural and • Technological features
12.	Tran et al. 2013	Analyzing the sources and nature of influence: how the Avahan program used evidence to influence HIV/AIDS prevention policy in India	Conceptual framework	HIV/AIDS; India	Bill & Melinda Gates Foundation	No information available
13.	Bertone et al. 2013	Assessing communities of practice in health policy: a conceptual framework as a first step towards empirical research	A simplified graphic representation of the conceptual framework for assessing communities of practice in health policy	Global health; African countries	Seventh Framework Programme of the European Commission, UNICEF Western and Central Africa Regional Office	Other contextual factors (including other knowledge management activities or other sources of knowledge)
14.	Timotijevic et al. 2013	EURRECA—A Framework for Considering Evidence in Public Health Nutrition Policy Development	Public Health Nutrition Policy-making Framework	Micronutrient dietary reference values; European countries	Commission of the European Communities, Specific Research Technology and Development (RTD) Programme Quality of Life and Management of Living Resources within the 6th framework programme	Wider context: • Global trends • Data, media • Broader consumer beliefs • Ethical considerations • Wider social, legal, political, and economic environment
15.	Onwujekwe et al. 2015	Role and use of evidence in policymaking: an analysis of case studies from the health sector in Nigeria	Framework for assessing the role of evidence in policy development	Maternal neonatal and child health, oral health, human resource for health; Nigeria	European Union 7th Framework Programme	No information available
16.	Redman et al. 2015	The SPIRIT Action Framework: A structured approach to selecting and testing strategies to increase the use of research in policy	SPIRIT Action Framework	Not specified; not specified	Australian National Health and Medical Research Council Centre of Research Excellence	Policy influences: • Public opinion • Media • Economic climate • Legislative/policy infrastructure • Political ideology and priorities • Stakeholder interests • Expert advice • Resources
17.	Spicer et al. 2016	‘The stars seem aligned’: a qualitative study to understand the effects of context on scale-up of maternal and newborn health innovations in Ethiopia, India and Nigeria	Analytic framework: contextual barriers and enablers to scale-up (of maternal and newborn health innovations)	Maternal and newborn health innovations; Ethiopia, Nigeria and India	Bill & Melinda Gates Foundation	Contextual factors influencing government decisions to accept, adopt and finance innovations at scale: How health policies are made • Government willingness to collaborate with development partners and implementers • Government responsiveness to civil society • Evidence-based decision making • Turnaround of government officials • Bureaucratic government institutions Prioritising and funding maternal and newborn health • National policy frameworks • Economic resources and global and development partners’ influence • Influence of powerful country actors Development partner harmonisation • Information sharing and coordinated communication with government • Embracing donor coordination mechanisms
18.	Bragge et al. 2017	AIMD—a validated, simplified framework of interventions to promote and integrate evidence into health practices, systems, and policies	AIMD (Aims, Ingredients, Mechanism, Delivery) framework	Not specified; not specified	KT Canada and The Canadian Institutes for Health Research	No information available
19.	Mulvale et al. 2017	Teasing apart “the tangled web” of influence of policy dialogues: lessons from a case study of dialogues about healthcare reform options for Canada	The dialogue to policy – web of influence	Healthcare Financing and Transformation; not specified	Canada; McMaster University Arts Research Board and Canadian Foundation for Healthcare Improvement	No information available
20.	Sarkies et al. 2017	The effectiveness of research implementation strategies for promoting evidence-informed policy and management decisions in healthcare: a systematic review	Conceptualisation of inter-related themes (analytic themes) associated with effective strategies and the inter-relationship between these factors	Not specified; not specified	None declared	No information available
21.	Houngbo et al. 2017	A Model for Good Governance of Healthcare Technology Management in the Public Sector: Learning from Evidence-Informed Policy Development and Implementation in Benin	A model for evidence-informed policy making, utilizing the perceptions of state and non-state actors to improve healthcare technology management	Healthcare Technology Management; Benin	Netherlands Organization for International Cooperation in Higher Education (NUFFIC)	No information available
22.	Mwendera et al. 2017	Development of a framework to improve the utilisation of malaria research for policy development in Malawi	Framework to promote the utilisation of malaria research for policy development in Malawi	Malaria; Malawi	University of Pretoria Institute for Sustainable Malaria Control and South African Medical Research Council	Contextual setting: Ministry of Health (MoH) • Political set up • Leadership system within the MoH • Government policies • Cultural set up
23.	Ellen et al. 2017	A Knowledge Translation framework on ageing and health	Framework for Knowledge Translation in ageing and health	Ageing; LMICs	AgeUK (a UK-based charity for older people)	Context and climate for ageing and health • Is there a willingness to accept ageing and health as an issue? Are there policies related to ageing and health or do existing health policies relate to ageing? Are they accessible? • Are there existing intermediary organizations on ageing and health? Is there a position or department in the government that supports ageing in general and ageing and health? Context and climate for knowledge translation • Is there an appetite or an interest for the use of evidence in policymaking? Do researchers and users understand the importance of Knowledge Translation in ageing and health? • Do leaders within the health systems promote the use of evidence in policymaking? • Do funders within the health systems have a mandate to support efforts to link research to action and do they support these efforts in several ways?
24.	Ongolo-Zogo et al. 2018	Assessing the influence of knowledge translation platforms on health system policy processes to achieve the health millennium development goals in Cameroon and Uganda: a comparative case study	Logical framework for KTP (Knowledge Translation Platform) influence	MDGs, malaria control, mother and child health; Cameroon and Uganda	International Research Chair Initiative in Evidence-Informed Health Policies, the Canadian Global Health Research Initiative	Contextual factors: • Institutions (structures, legacies, policy networks) • Interests • Ideas (values, research evidence) • External factors (reports, commitments)
25.	Plamondon et al. 2019	Blending integrated knowledge translation with global health governance: an approach for advancing action on a wicked problem	Blending processes and mechanisms for a blended integrated knowledge translation (IKT) – global health governance (GHG) approach	Global health; Global South	Integrated Knowledge Translation Network, funded by a Canadian Institutes of Health Research Foundation Grant	Moments in the IKT cycle • Adapt knowledge to local context Complementary GHG processes and mechanisms • Consideration of the composition of non-traditional actors, such as civil society and private sector, in governance bodies • Guidance for meaningful engagement between actors, particularly in shared governance models
26.	Vincenten et al. 2019	Factors Influencing Implementation of Evidence-based Interventions in Public Health Systems – a Model	Evidence implementation model for public health systems	Not specified; not specified	None declared	Context setting
27.	Motani et al. 2019	Lessons learned from Evidence-Informed Decision-Making in Nutrition & Health (EVIDENT) in Africa: a project evaluation	EVIDENT’s conceptual framework for evidence-informed decision-making	Nutrition; African countries	Belgian Development Cooperation	Contextualising evidence
28.	Varallyay et al. 2020	Health system decision-makers at the helm of implementation research: development of a framework to evaluate the processes and effectiveness of embedded approaches	Working conceptual model for embedded implementation research	Not specified; Latin America and the Caribbean	Evidence and Intelligence for Action in Health Department of the Pan American Health Organization, in partnership with the Alliance for Health Policy and Systems Research at WHO	Context • Type of programme/policy under study • The perceived value of evidence in decision-making circles • Political will and support for the improvement of the targeted programme (i.e. relative priority) • Health system governance, namely decentralisation of decision-making, staff turnover and regulatory structures or institutional incentives • Political stability and underlying political interests • Access to external technical assistance for IR • Availability of funding for research as well as post-research activities
29.	Leonard et al. 2020	Barriers and facilitators to implementing evidence-based health innovations in low- and middle-income countries: A systematic literature review	Cause-effect diagram of health innovation implementation in LMICs, Context sub-components	Health innovation; LMICs	Janssen Pharmaceutical	• Political: the leadership in the ministry of health; ideological beliefs; the presence of politicised issues such as abortion, homosexuality or prostitution; the presence of corruption; conflict, which can lead to unregulated markets, uncoordinated aid work, and security issues; and the political climate, internationally and locally • Environment: the distribution of health facilities; the distance between facilities and between the communities being served by a facility; the remoteness of a facility; the transportation systems linking health facilities to each other and to communities; the type of terrain present where the innovation is being implemented; the weather conditions, including the humidity levels, temperatures and the presence of natural disasters; and the altitude and levels of dust in the environment that the innovation is being implemented • Socio-cultural: social and gender norms, cultural beliefs such as satanism and witchcraft, religious beliefs, the historical contexts (such as the presence of exploited or marginalized populations), and traditional or indigenous health practices • Socio-demographic: the languages spoken, immigration status, literacy of the population, age groups, and employment status • Economic: the economic stability and status of an area, as well as community, national and international financial constraints • Epidemiology: competing health priorities which are a result of the different diseases present in a country
30.	Votruba et al. 2020	The EVITA framework for evidence-based mental health policy agenda setting in low- and middle-income countries	The validated EVITA 1.1 framework	Mental health; LMICs	National Institute for Health Research Collaboration for Leadership in Applied Health Research and Care South London at King’s College Hospital NHS Foundation Trust	• External influences: socioeconomic context, culture, societal values and beliefs relevant to forces and impulses on the issue, from outside policymaking (political context) or evidence generator sphere • Political context: the sum of national politics, policy and polity structures, institutions, mechanisms and policymaking processes. This includes power distribution, (in)formal rules, political will, interests, motives and opportunities of people and organizations involved
31.	Votruba et al. 2021	EVITA 2.0, an updated framework for understanding evidence-based mental health policy agenda-setting: tested and informed by key informant interviews in a multilevel comparative case study	The new EVITA 2.0 framework	Mental health; LMICs	National Institute for Health Research Collaboration for Leadership in Applied Health Research and Care South London at King’s College Hospital NHS Foundation Trust	• External context • Policy sphere, encompassing policy agenda, window of opportunity, political will and key individuals
32.	Kuchenmüller et al. 2022	A comprehensive monitoring and evaluation framework for evidence to policy networks	EVIPNet Europe Theory of Change	Not specified	WHO Europe	Contextual factors for evidence-informed policymaking: • Political • Economic • Logistic • Administrative

Notably, the first three publications in our scoping review are authored by scholars affiliated with Canada, which is less of a coincidence, given the role of Canadian Institutes of Health Research (CIHR), the federal funding agency for health research: The CIHR Act (Bill C-13) mandates CIHR to ensure that the translation of health knowledge permeates every aspect of its work [48]. Moreover, it was CIHR that coined the term Knowledge Translation, defining KT as ‘a dynamic and iterative process that includes the synthesis, dissemination, exchange and ethically sound application of knowledge to improve health, provide more effective health services and products, and strengthen the health care system’ [49]*.* This comprehensive definition has since been adapted by international organisations (IOs), including WHO. The first document published by WHO that utilised KT to influence health policies dates back to 2005, entitled ‘Bridging the “know-do” gap: Meeting on knowledge translation in global health’, an initiative that was supported by the Canadian Coalition for Global Health Research, the Canadian International Development Agency, the German Agency for Technical Cooperation and the WHO Special Programme on Research and Training in Tropical Diseases [1]. Following this official recognition by WHO, studies in our scoping review after 2005 indicate a noticeable expansion of KT, encompassing a wider geographical area than Canada.

The article of Ashford et al. from 2006 [50] discusses the challenge of policy decisions in Kenya in the health field being disconnected from scientific evidence and presents a model for translating knowledge into policy actions through agenda-setting, coalition building and policy learning. However, the framework lacks explicit incorporation of contextual factors influencing health policies. Bauman et al. [51] propose a six-step framework for successful dissemination of physical activity evidence, illustrated through four case studies from three countries (Canada, USA and Brazil) and a global perspective. They interpret contextual factors as barriers and facilitators to physical activity and public health innovations. Focusing on the USA, Gold [52] explains factors, processes and actors that shape pathways between research and its use in a summary diagram, including a reference to ‘other influences in process’ for context. Green et al. [4] examine the gap between health research and its application in public health without focusing on a specific geographical area. Their study comprehensively reviews various concepts of diffusion, dissemination and implementation in public health, proposing ways to blend diffusion theory with other theories. Their ‘utilization-focused surveillance framework’ interprets context as social determinants as structures, economics, politics and culture.

Further, the article by Dhonukshe-Rutten et al. from 2010 [53] presents a general framework that outlines the process of translating nutritional requirements into policy applications from a European perspective. The framework incorporates scientific evidence, stakeholder interests and the socio-political context. The description of this socio-political context is rather brief, encompassing political and social priorities, legal context, ethical issues and economic implications. Ir et al. [54] analyse the use of knowledge in shaping policy on health equity funds in Cambodia, with the objective of understanding how KT contributes to the development of health policies that promote equity. Yet no information on context is available in the framework that they suggest. A notable exception among these early KT TMFs until 2010 is the conceptual framework for analysing integration of targeted health interventions into health systems by Atun et al. [55], in which the authors provide details about the factors that have an influence on the process of bringing evidence to health policies. Focusing on the adoption, diffusion and assimilation of health interventions, their conceptual framework provides a systematic approach for evaluating and informing policies in this field. Compared to the previous studies discussed above, their definition of context for this framework is comprehensive (Table 2). Overall, most of the studies containing macro-level KT TMFs published until 2010 either do not fully acknowledge contextual factors or provide generic terms such as cultural, political and economic for brief description (9 out of 10; 90%).

Studies published after 2010 demonstrate a notable geographical shift, with a greater emphasis on low- and middle-income countries (LMICs). By taking the adoption of the directly observed treatment, short-course (DOTS) strategy for tuberculosis control in Mexico as a case study, Bissell et al. [56] examine policy transfer to Mexico and its relevance to operational research efforts and suggest a model for analysis of health policy transfer. The model interprets context as health system, including political, economic, social, cultural and technological features. Focusing on HIV/AIDS in India, Tran et al. [57] explore KT by considering various forms of evidence beyond scientific evidence, such as best practices derived from programme experience and disseminated through personal communication. Their proposed framework aims to offer an analytical tool for understanding how evidence-based influence is exerted. In their framework, no information is available on context. Next, Bertone et al. [58] report on the effectiveness of Communities of Practice (CoPs) in African countries and present a conceptual framework for analysing and assessing transnational CoPs in health policy. The framework organises the key elements of CoPs, linking available resources, knowledge management activities, policy and practice changes, and improvements in health outcomes. Context is only briefly included in this framework.

Some other studies include both European and global perspectives. The publication from Timotijevic et al. from 2013 [59] introduces an epistemological framework that examines the considerations influencing the policy-making process, with a specific focus on micronutrient requirements in Europe. They present case studies from several European countries, highlighting the relevance of the framework in understanding the policy context related to micronutrients. Context is interpreted in this framework as global trends, data, media, broader consumer beliefs, ethical considerations, and wider social, legal, political, and economic environment. Next, funded by the European Union, the study by Onwujekwe et al. [60] examines the role of different types of evidence in health policy development in Nigeria. Although they cover the factors related to policy actors in their framework for assessing the role of evidence in policy development, they provide no information on context. Moreover, Redman et al. [61] present the SPIRIT Action Framework, which aims to enhance the use of research in policymaking. Context is interpreted in this framework as policy influences, i.e. public opinion, media, economic climate, legislative/policy infrastructure, political ideology and priorities, stakeholder interests, expert advice, and resources. From a global perspective, Spicer et al. [62] explore the contextual factors that influenced the scale-up of donor-funded maternal and newborn health innovations in Ethiopia, India and Nigeria, highlighting the importance of context in assessing and adapting innovations. Their suggested contextual factors influencing government decisions to accept, adopt and finance innovations at scale are relatively comprehensive (Table 2).

In terms of publication frequency, the pinnacle of reviewed KT studies was in 2017. Among six studies published in 2017, four lack details about context in their KT conceptualisations and one study touches on context very briefly. Bragge et al. [5] brought for their study an international terminology working group together to develop a simplified framework of interventions to integrate evidence into health practices, systems, and policies, named as the Aims, Ingredients, Mechanism, Delivery framework, albeit without providing details on contextual factors. Second, Mulvale et al. [63] present a conceptual framework that explores the impact of policy dialogues on policy development, illustrating how these dialogues can influence different stages of the policy cycle. Similar to the previous one, this study too, lacks information on context. In a systematic review, Sarkies et al. [64] evaluate the effectiveness of research implementation strategies in promoting evidence-informed policy decisions in healthcare. The study explores the factors associated with effective strategies and their inter-relationship, yet without further information on context. Fourth, Houngbo et al. [65] focus on the development of a strategy to implement a good governance model for health technology management in the public health sector, drawing from their experience in Benin. They outline a six-phase model that includes preparatory analysis, stakeholder identification and problem analysis, shared analysis and visioning, development of policy instruments for pilot testing, policy development and validation, and policy implementation and evaluation. They provide no information about context in their model. Fifth, Mwendera et al. [66] present a framework for improving the use of malaria research in policy development in Malawi, which was developed based on case studies exploring the policymaking process, the use of local malaria research, and assessing facilitators and barriers to research utilisation. Contextual setting is considered as Ministry of Health (MoH) with political set up, leadership system within the MoH, government policies and cultural set up. In contrast to these five studies, Ellen et al. [67] present a relatively comprehensive framework to support evidence-informed policymaking in ageing and health. The framework includes thought-provoking questions to discover contextual factors (Table 2).

Continuing the trend, studies published after 2017 focus increasingly on LMICs. In their embedded case study, Ongolo-Zogo et al. [68] examine the influence of two Knowledge Translation Platforms (KTPs) on policy decisions to achieve the health millennium development goals in Cameroon and Uganda. It explores how these KTPs influenced policy through interactions within policy issue networks, engagement with interest groups, and the promotion of evidence-supported ideas, ultimately shaping the overall policy climate for evidence-informed health system policymaking. Contextual factors are thereby interpreted as institutions (structures, legacies, policy networks), interests, ideas (values, research evidence) and external factors (reports, commitments). Focusing on the ‘Global South’, Plamondon et al. [69] suggest blending integrated knowledge translation with global health governance as an approach for strengthening leadership for health equity action. In terms of contextual factors, they include some information such as adapting knowledge to local context, consideration of the composition of non-traditional actors, such as civil society and private sector, in governance bodies and guidance for meaningful engagement between actors, particularly in shared governance models. Further, Vincenten et al. [70] propose a conceptual model to enhance understanding of interlinking factors that influence the evidence implementation process. Their evidence implementation model for public health systems refers to ‘context setting’, albeit without providing further detail.

Similarly, the study by Motani et al. from 2019 [71] assesses the outcomes and lessons learned from the EVIDENT partnership that focused on knowledge management for evidence-informed decision-making in nutrition and health in Africa. Although they mention ‘contextualising evidence’ in their conceptual framework, information about context is lacking. Focusing on Latin America and the Caribbean, Varallyay et al. [72] introduce a conceptual framework for evaluating embedded implementation research in various contexts. The framework outlines key stages of evidence-informed decision-making and provides guidance on assessing embeddedness and critical contextual factors. Compared to others, their conceptual framework provides a relatively comprehensive elaboration on contextual factors. In addition, among all the studies reviewed, Leonard et al. [73] present an exceptionally comprehensive analysis, where they identify the facilitators and barriers to the sustainable implementation of evidence-based health innovations in LMICs. Through a systematic literature review, they scrutinise 79 studies and categorise the identified barriers and facilitators into seven groups: context, innovation, relations and networks, institutions, knowledge, actors, and resources. The first one, context, contains rich information that could be seen in Table 2.

Continuing from LMICs, Votruba et al. [74] present in their study the EVITA (EVIdence To Agenda setting) conceptual framework for mental health research-policy interrelationships in LMICs with some information about context, detailed as external influences and political context. In a follow-up study, they offer an updated framework for understanding evidence-based mental health policy agenda-setting [75]. In their revised framework, context is interpreted as external context and policy sphere, encompassing policy agenda, window of opportunity, political will and key individuals. Lastly, to develop a comprehensive monitoring and evaluation framework for evidence-to-policy networks, Kuchenmüller et al. [76] present the EVIPNet Europe Theory of Change and interpret contextual factors for evidence-informed policymaking as political, economic, logistic and administrative. Overall, it can be concluded that studies presenting macro-level KT TMFs from 2011 until 2022 focus mainly on LMICs (15 out of 22; close to 70%) and the majority of them were funded by international (development) organisations, the European Commission and global health donor agencies. An overwhelming number of studies among them (19 out of 22; close to 90%) provide either no information on contextual details or these were included only partly with some generic terms in KT TMFs.

## Discussion

Our systematic scoping review suggests that the approach of KT, which has evolved from evidence-based medicine to evidence-informed policymaking, tends to remain closely tied to its clinical origins when developing TMFs. In other words, macro-level KT TMFs place greater emphasis on the (public) health issue at hand rather than considering the broader decision-making context, a viewpoint shared by other scholars as well [30]. One reason could be that in the early stages of KT TMFs, the emphasis primarily focused on implementing evidence-based practices within clinical settings. At that time, the spotlight was mostly on content, including aspects like clinical studies, checklists and guidelines serving as the evidence base. In those meso-level KT TMFs, a detailed description of context, i.e. the overall environment in which these practices should be implemented, might have been deemed less necessary, given that healthcare organisations, such as hospitals to implement medical guidelines or surgical safety checklists, show similar characteristics globally.

However, as the scope of KT TMFs continues to expand to include the influence on health policies, a deeper understanding of context-specific factors within different jurisdictions and the dynamics of the policy process is becoming increasingly crucial. This is even more important for KT scholars aiming to conceptualise large-scale changes, as described in KT Tier 5, which necessitate a thorough understanding of targeted behaviours within societies. As the complexity of interventions increases due to the growing number of stakeholders either affecting or being affected by them, the interventions are surrounded by a more intricate web of attitudes, incentives, relationships, rules of engagement and spheres of influence [7]. The persisting emphasis on content over context in the evolving field of KT may oversimplify the complex process of using evidence in policymaking and understanding the society [77]. Some scholars argue that this common observation in public health can be attributed to the dominance of experts primarily from medical sciences [78–80]. Our study confirms the potential limitation of not incorporating insights from political science and public policy studies, which can lead to what is often termed a ‘naïve’ conceptualisation of evidence-to-policy schemes [15–17]. It is therefore strongly encouraged that the emerging macro-level KT concepts draw on political science and public administration if KT scholars intend to effectively communicate new ideas to policymakers, with the aim of prompting their action or response. We summarised our findings into three points.

Firstly, KT scholars may want to identify and pinpoint exactly where a change should occur within the policy process. The main confusion that we observed in the KT literature arises from a lack of understanding of how public policies are made. Notably, the term ‘evidence-informed policymaking’ can refer to any stage of the policy cycle, spanning from agenda-setting to policy formulation, adoption, implementation and evaluation. Understanding these steps will allow researchers to refine their language when advocating for policy changes across various jurisdictions; for instance, the word ‘implementation’ is often inappropriately used in KT literature. As commonly known, at the macro-level, public policies take the form of legislation, law-making and regulation, thereby shaping the practices or policies to be implemented at the meso- and micro-levels [81]. In other words, the process of using specific knowledge to influence health policies, however evidence-based it might be, falls mostly under the responsibility and jurisdiction of sovereign states. For this reason, macro-level KT TMFs should reflect the importance of understanding the policy context and the complexities associated with policymaking, rather than suggesting flawed or unrealistic top-down ‘implementation’ strategies in countries by foregrounding the content, or the (public) health issue at hand.

Our second observation from this systematic scoping review points towards a selective perception among researchers when reporting on policy interventions. Research on KT does not solely exist due to the perceived gap between scientific evidence and policy but also because of the pressures the organisations or researchers face in being accountable to their funding sources, ensuring the continuity of financial support for their activities and claiming output legitimacy to change public policies [8]. This situation indirectly compels researchers working to influence health policies in the field to provide ‘evidence-based’ feedback on the success of their projects to donors [82]. In doing so, researchers may overly emphasise the content of the policy intervention in their reporting to secure further funding, while they underemphasis the contextual factors. These factors, often perceived as a given, might actually be the primary facilitators of their success. Such a lack of transparency regarding the definition of context is particularly visible in the field of global health, where LMICs often rely on external donors. It is important to note that this statement is not intended as a negative critique of their missions or an evaluation of health outcomes in countries following such missions. Rather, it seeks to explain the underlying reason why researchers, particularly those reliant on donors in LMICs, prioritise promoting the concept of KT from a technical standpoint, giving less attention to contextual factors in their reasoning.

Lastly, and connected to the previous point, it is our observation that the majority of macro-level KT TMFs fail to give adequate consideration to both power dynamics in countries (internal vs. external influences) and the actual role that government plays in public policies. Notably, although good policymaking entails an honest effort to use the best available evidence, the belief that this will completely negate the role of power and politics in decision-making is a technocratic illusion [83]. Among the studies reviewed, the framework put forth by Leonard et al. [73] offers the most comprehensive understanding of context and includes a broad range of factors (such as political, social, and economic) discovered also in other reviewed studies. Moreover, the framework, developed through an extensive systematic review, offers a more in-depth exploration of these contextual factors than merely listing them as a set of keywords. Indeed, within the domains of political science and public policy, such factors shaping health policies have received considerable scholarly attention for decades. To define what context entails, Walt refers in her book ‘Health Policy: An Introduction to Process and Power’ [84] to the work of Leichter from 1979 [85], who provides a scheme for analysing public policy. This includes i) situational factors, which are transient, impermanent, or idiosyncratic; ii) structural factors, which are relatively unchanging elements of the society and polity; iii) cultural factors, which are value commitments of groups; and iv) environmental factors, which are events, structures and values that exist outside the boundaries of a political system and influence decisions within it. His detailed sub-categories for context can be found in Table 3. This flexible public policy framework may offer KT researchers a valuable approach to understanding contextual factors and provide some guidance to define the keywords to focus on. Scholars can adapt this framework to suit a wide range of KT topics, creating more context-sensitive and comprehensive KT TMFs.

**Table 3:** Contextual factors influencing public policies, adapted from Leichter [85]

Situational factors A. Violent events, e.g. international and civil wars B. Economic cycles, e.g. inflation C. Natural disasters, e.g. epidemics D. Political events and conditions i. Political status change ii. Political regime change iii. Change of government iv. Political reform v. Political corruption or scandal vi. Change in political leadership E. Technological change F. The policy agenda; competition among policy issues
Structural factors A. Political structure i. Type of political regime, e.g. military or civilian ii. Type of political organisation (federal or unitary system) iii. Form of government (parliamentary, presidential, nondemocratic) iv. Group activity (number, strength, and legitimacy of interest groups) v. Political process, e.g. nature of bureaucracy vi. Policy constraints (incrementalism, prior policy commitments) B. Economic structure i. Type of economic system (free market, planned, or mixed economy) ii. Economic base, e.g. primarily agrarian or industrial iii. National wealth and income, e.g. distribution of wealth iv. Complexity of economic organisation (modern or traditional economy) C. Social, demographic, and ecological structure i. Population, e.g. age structure, birth rate and level of education ii. Degree of urbanisation, e.g. proportion of population living in urban and rural areas iii. Natural resources (land, water, minerals) iv. Geographic location, e.g. island or landlocked
Cultural factors A. Political culture i. National heritage ii. Political norms and values (the role of the individual and the state) iii. Formal political ideology B. General culture i. Traditional social values (relating to social institutions and values such as marriage, the family, sex roles) ii. Religion (religious values and role of religious institutions in society)
Environmental factors A. International political environment, e.g. cold war B. Policy diffusion (emulation and borrowing of policy ideas and solutions from other nations) C. International agreements, obligations, and pressures i. World public opinion ii. International affiliations, e.g. United Nations iii. Participation in international conferences and agreements iv. International financial obligations, e.g. World Bank loans D. International private corporations

Admittedly, our study has certain limitations. Despite choosing one of the most comprehensive bibliographic databases for our systematic scoping review, which includes materials from biomedicine, allied health fields, biological and physical sciences, humanities, and information science in relation to medicine and healthcare, we acknowledge that we may have missed relevant articles indexed in other databases. Hence, exclusively using Ovid/MEDLINE due to resource constraints may have narrowed the scope and diversity of scholarly literature examined in this study. Second, our review was limited to peer-reviewed publications in English and German. Future studies could extend our findings by examining the extent to which contextual factors are detailed in macro-level KT TMFs published in grey literature and in different languages. Given the abundance of KT reports, working papers or policy briefs published by IOs and development agencies, such an endeavour could enrich our findings and either support or challenge our conclusions. Nonetheless, to our knowledge, this study represents the first systematic review and critical appraisal of emerging knowledge-to-policy concepts, also known as macro-level KT TMFs. It successfully blends insights from both biomedical and public policy disciplines, and could serve as a roadmap for future research.

## Conclusion

The translation of knowledge to policymakers involves more than technical skills commonly associated with (bio-)medical sciences, such as creating evidence-based guidelines or clinical checklists. Instead, evidence-informed policymaking reflects an ambition to engage in the political dimensions of states. Therefore, the evolving KT concepts addressing health policies should be seen as a political decision-making process, rather than a purely analytical one, as is the case with evidence-based medicine. To better understand the influence of power dynamics and governance structures in policymaking, we suggest that future macro-level KT TMFs draw on insights from political science and public administration. Collaborative, interdisciplinary research initiatives could be undertaken to bridge the gap between these fields. Technocratic KT TMFs that overlook contextual factors risk propagating misconceptions in academic circles about how health policies are made, as they become increasingly influential over time. Research, the systematic pursuit of knowledge, is neither inherently good nor bad; it can be sought after, used or misused, like any other tool in policymaking. What is needed in the KT discourse is not another generic call for ‘research-to-action’ but rather an understanding of the dividing line between research-to-*clinical*-action and research-to-*political*-action.

## Data Availability

Available upon reasonable request.
